# Association of Macular Thickness With Age and Age-Related Macular Degeneration in the Carotenoids in Age-Related Eye Disease Study 2 (CAREDS2), An Ancillary Study of the Women's Health Initiative

**DOI:** 10.1167/tvst.10.2.39

**Published:** 2021-02-25

**Authors:** Tyler Etheridge, Zhe Liu, Marine Nalbandyan, Spencer Cleland, Barbara A. Blodi, Julie A. Mares, Steven Bailey, Robert Wallace, Karen Gehrs, Lesley F. Tinker, Ronald Gangnon, Amitha Domalpally, for the CAREDS2 Research Study Group

**Affiliations:** 1 Department of Ophthalmology and Visual Sciences, University of Wisconsin School of Medicine and Public Health, Madison, WI, USA; 2 Oregon Health Sciences University Casey Eye Institute, Portland, OR, USA; 3 University of Iowa, College of Public Health, Department of Epidemiology, Iowa City, IA, USA; 4 University of Iowa, Department of Ophthalmology, University of Iowa, Iowa City, IA, USA; 5 Cancer Research Program, Public Health Sciences Division, Fred Hutchinson Cancer Research Center, Seattle, WA, USA; 6 Department of Population Health Sciences, University of Wisconsin School of Medicine and Public Health, Madison, WI, USA; 7 Department of Biostatistics & Medical Informatics, University of Wisconsin-Madison, Madison, WI, USA

**Keywords:** age-related macular degeneration, carotenoids in age-related eye disease study 2, macular thickness, optical coherence tomography, women's health initiative, postmenopausal, women

## Abstract

**Purpose:**

To evaluate the relationship of retinal layer thickness with age and age-related macular degeneration (AMD) in the Carotenoids in Age-Related Eye Disease Study 2.

**Methods:**

Total retinal thickness within the macular area, and individual layer thickness was determined for CAREDS2 participants (n = 906 eyes, 473 women) from the Women's Health Initiative using Heidelberg optical coherence tomography (OCT). Mean measurements within the OCT grid were compared across age tertiles (69–78, 78–83, and 83–101 years) and AMD outcomes.

**Results:**

Mean retinal thickness in the central circle, inner ring, and outer ring were 277 ± 34 µm, 326 ± 20 µm, and 282 ± 15 µm, respectively. Thickness did not vary by age in the central circle, but decreased with age in the inner and outer circles (*P* ≤ 0.004). Specifically, ganglion cell (GCL), inner plexiform, and outer nuclear (ONL) layer thickness decreased with age (*P* ≤ 0.003). Age-adjusted retinal thickness in all three circles did not vary by AMD outcomes (486 without AMD and 413 with AMD). However, individual layers showed changes with GCL and photoreceptor thinning and retinal pigment epithelial thicknening in eyes with late AMD. After controlling for age and AMD, higher ONL thickness was associated with better visual acuity.

**Conclusions:**

In this cohort of older women, a decrease in perifoveal thickness was associated with increasing age, particularly in the inner retinal layers. Variabilty in thickness in AMD eyes was primarily due to outer retinal layers. Among all retinal layers, the ONL plays an important role in preserving visual acuity.

**Translational Relevance:**

The study provides a deeper understanding of age related changes to the retinal layers and their effect on visual loss.

## Introduction

Optical coherence tomography (OCT) provides noninvasive in vivo cross-sectional imaging of the retina. Spectral domain OCT (SD-OCT) devices permit the demarcation of individual retinal layers at higher acquisition speeds and image resolution from previous devices.^1^ In addition, SD-OCT enables automated segmentation and quantification of individual retinal layers through the application of various software and segmentation algorithms. Previous studies have demonstrated that SD-OCT segmentation software produces reproducible thickness measurements.^2^

SD-OCT is used extensively in clinical practice and clinical trials to measure the thickness of the retina and identify anatomic changes that occur with diseases, including those related to aging. For example, in age-related macular degeneration (AMD), SD-OCT segmentation has been used to monitor drusen, choroidal neovascularization, and geographic atrophy.^3^ SD-OCT segmentation is also used to identify thinning of the nerve fiber layer in neurodegenerative diseases, such as Alzheimer's disease.^4^ The description of SD-OCT changes in diseases of aging have been constrained by small sample sizes of populations younger than 75 years and an overrepresentation of severe disease. In addition, normative thickness data from proprietary SD-OCT manufacturers are not publicly available.

Previous studies that have used SD-OCT segmentation to evaluate the distribution of retinal layer thickness measurements across population subgroups were vital to understanding the epidemiological associations and pathophysiological mechanisms of disease, as well as to define normal ranges.^5^^–^^12^ However, these studies have been limited to population studies without retinal diseases. We used data from the Carotenoids in Age-Related Eye Disease Study 2 (CAREDS2), a unique, and well-characterized cohort of women aged 69 to 101 years at the time of their participation in 2016 to 2019, to provide the macular thickness measurements and to study the relationship with age and AMD. We also explored relationships between the thickness of individual retinal layers and best-corrected visual acuity. CAREDS2 is an ancillary study of the Women's Health Initiative Observation Study (WHI-OS).

## Methods

### Participants

The details of the CAREDS study population has been previously published.^13^ In summary, the WHI-OS, a prospective cohort study at 40 sites throughout the United States investigating the most common causes of morbidity and mortality among postmenopausal women aged 50 to 79 years (n = 3143).^14^ Eligibility criteria included having high or low dietary lutein plus zeaxanthin intake levels (>78th or <28th percentiles) at WHI enrollment (1994–1998). Women from three WHI-OS study sites were invited to participate in CAREDS: the University of Wisconsin (Madison, WI), the University of Iowa (Iowa City, IA), and the Kaiser Center for Health Research (Portland, OR), were recruited for CAREDS baseline (2001–2004) (n = 2005). Of the CAREDS cohort, 685 participated in the CAREDS2 follow-up (2016–2019), of which 487 participated in study visits in-person. The analysis dataset for this project includes 473 women who completed SD-OCT scans. The age of the participants at the time of OCT assessment in CAREDS2 ranged from 69 to 101 years. The study was approved by the institutional review boards associated with each center. All participants provided written informed consent, and the study adhered to the tenets of the Declaration of Helsinki.

### SD-OCT Imaging

SD-OCT scans were acquired by certified photographers with a Heidelberg Spectralis (Heidelberg Engineering, Heidelberg, Germany) OCT machine following the CAREDS2 reading center (Fundus Photograph Reading Center, University of Wisconsin–Madison) approved protocol. Volume scans centered on the macula were obtained from both eyes for all study participants. The macular scan protocol includes a 20° × 20° volume scan at high speed with 97 B scans, interscan distance of 60 µm and an image averaging of five frames.

### Retinal Layer Segmentation

**Figure 1.**
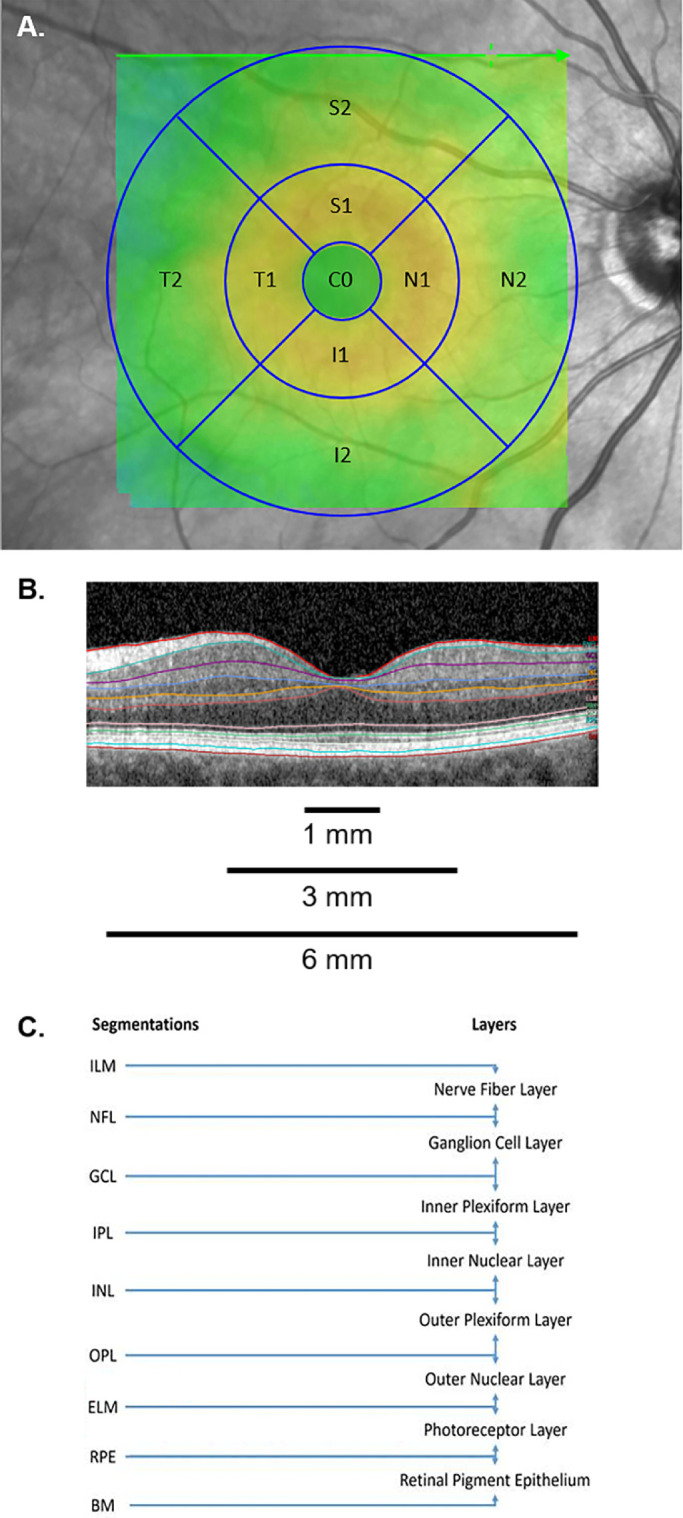
Representative SD-OCT scan and the nine sectors of the ETDRS grid with segmentation performed and retinal layers evaluated. (**A**) The nine sectors of the ETDRS grid, including the central circle cell (*C0*), inner nasal cell (*N1*), outer nasal cell (*N2*), inner superior cell (*S1*), outer superior cell (*S2*), inner temporal cell (*T1*), outer temporal cell (*T2*), inner inferior cell (*I1*), and outer inferior cell (*I2*). The nine sectors of the ETDRS grid were combined to form the central circle (1 mm diameter), inner ring (3 mm diameter), and outer ring (6 mm diameter). (**B**) Representative SD-OCT B-scan with default segmentation through the fovea, including the ILM (red), RNFL (teal), GCL (purple), IPL (blue), INL (orange), OPL (maroon), ONL (yellow), ELM (pink), PR (green), RPE (light blue), and BM (red). (**C**) Depiction of segmentation performed, and retinal layers evaluated.

Retinal layers were automatically segmented using Heidelberg Spectralis software (version 1.9.13.0). Layers were defined using the boundaries generated by the software. The layers, including internal limiting membrane (ILM), retinal nerve fiber layer, ganglion cell layer (GCL), inner plexiform layer (IPL), inner nuclear layer, outer plexiform layer, outer nuclear layer (ONL), photoreceptor layer (PR), and retinal pigment epithelium (RPE), are shown in Figure 1. The boundaries of the total retina were defined as ILM to BM. The total retina was divided into the inner and outer retinal layers by the software. The inner retinal layers were defined as ILM to external limiting membrane and the outer retinal layers as external limiting membrane to BM, respectively.

The segmentation was reviewed throughout the entire Early Treatment in Diabetic Retinopathy Study (ETDRS) grid and segmentation errors were manually edited within the Heidelberg Spectralis software in all scans as needed. Segmentation review and editing was performed by masked, study certified graders at the CAREDS2 reading center. A total of 946 SD-OCT images from 473 CAREDS2 study participants were available for segmentation. ETDRS grid centration was assessed and scans with additional artifacts, such as z-offset (n = 3) and B-scan inversion (n = 2) were excluded. Additional SD-OCT scans were excluded because of grossly inaccurate segmentation throughout all retinal layers beyond that correctable by manual editing (n = 2). The remainder of excluded SD-OCT scans were due to confounding pathology, including macular hole (n = 8), significant intraretinal or subretinal fluid (n = 4), and severe macular degeneration (n = 21) prohibiting the identification of individual retinal layers.

Thickness maps were generated within Heidelberg Spectralis software, providing mean macular thickness (µm) and volume (mm^3^) measurements for the nine sectors of the ETDRS grid, which were combined to form three concentric circles, including the central circle (1 mm diameter), inner ring (3 mm diameter), and outer ring (6 mm diameter) as displayed in Figure 1.

### AMD Assessment

AMD presence and severity at CAREDS baseline (2001–2004) was evaluated using 30° stereoscopic fundus photographs and at CAREDS2 follow-up (2016-2019) using 30° stereoscopic digital images. Of the 487 CAREDS2 participants, AMD status was determined from fundus photographs in 470 (96.5%) participants. AMD status of an additional five participants was determined from provider reports or medical records. Three field stereoscopic color photographs were evaluated for presence and severity of AMD using the Age-related Eye Disease Study (AREDS) severity scale.^15^ Based on CAREDS2 protocol defined outcomes, we grouped the original AREDS 12-step AMD scale into early AMD (AREDS levels 1–5), intermediate AMD (AREDS levels 6–8) and late AMD (AREDS levels 9–11b). Late AMD was further classified as geographic atrophy and neovascular AMD.

### Best Corrected Visual Acuity

Best corrected visual acuity (BCVA) was measured using the standardized ETDRS protocol modified for the AREDS trials.^16^

### Statistical Analysis

Macular thickness and volume measurements of the central circle (1 mm diameter), inner ring (3 mm diameter), and outer ring (6 mm diameter) were expressed as means ± standard deviation (SD) or least squares mean ± standard error (SE). Age was categorized into tertiles, and a *P* value for continuous trend over years was computed. AMD outcomes were expressed as a categorical variable, including no AMD, early AMD, intermediate AMD, and late AMD. We investigated the association between mean macular thickness and age tertile, as well as mean macular thickness and AMD outcomes, adjusted for age. Generalized estimating equations were used, which enabled the use of scans from both study eyes of each participant. Empirical standard error estimates were used for generalized estimating equations parameter estimation considering within person correlation. A two-tailed *P* value < 0.05 was considered significant. We also examined the age-adjusted associations between BCVA and individual layer thickness in the central circle, including sensitivity analyses, excluding participants with cataract and late AMD. Statistical analysis was performed using SAS software, version 9.4 (SAS Institute Inc., Cary, NC, USA).

## Results

### Macular Thickness by Age

**Figure 2.**
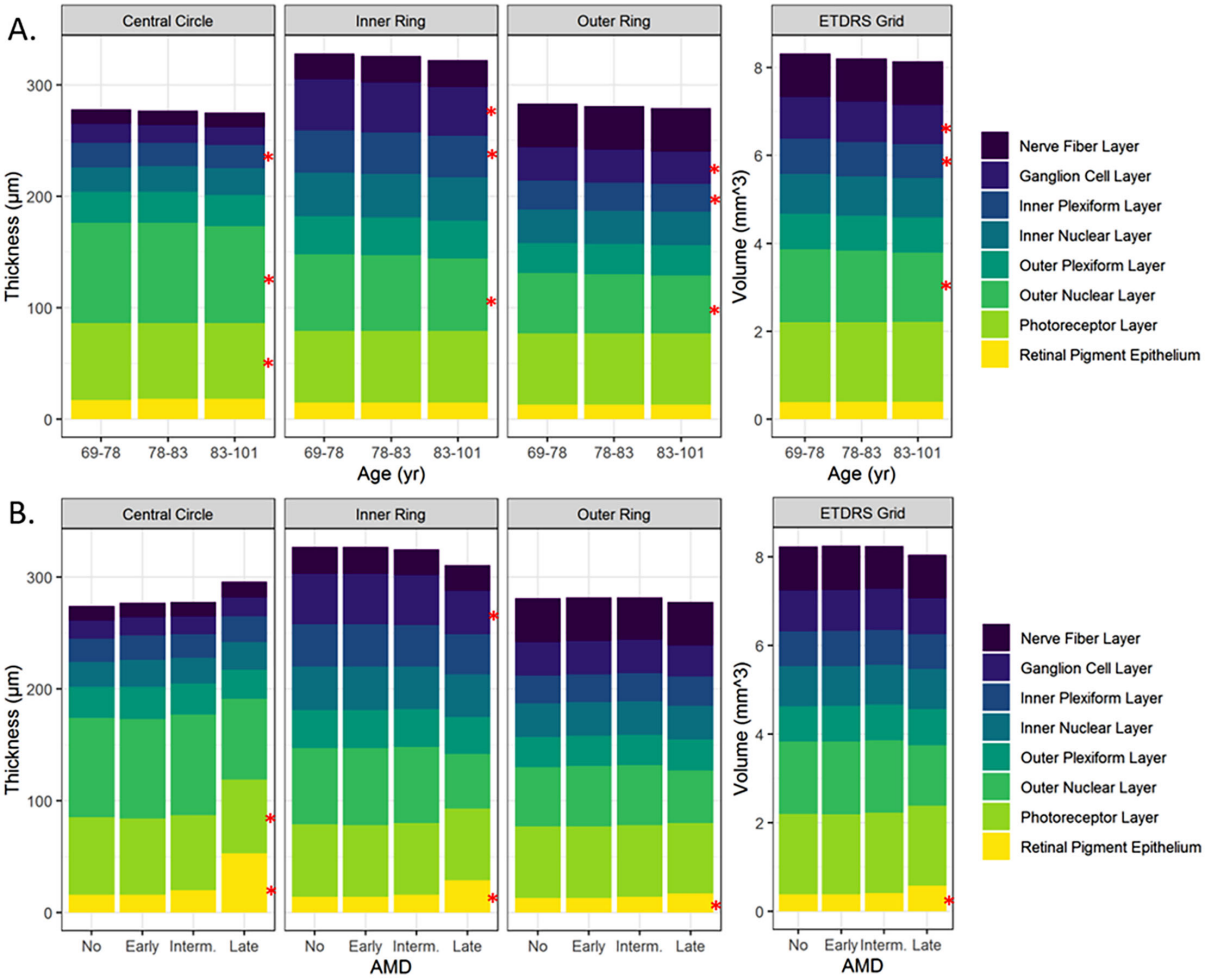
Mean macular thickness and volume by retinal layer. (**A**) Thickness and volume by age tertile (years). (**B**) Thickness and volume by AMD outcome, age adjusted. * indicates *P* < 0.05.

Overall, 946 SD-OCT scans of both eyes of 473 CAREDS2 participants were available for the current analysis. After excluding 40 scans, 906 scans were evaluated as presented in Table 1. Mean (SD) macular thickness overall, and in individual retinal layers are described by age tertile, within each of the nine ETDRS subfields (Fig. 2A and Table 2). In the central circle, we did not observe significant variations in the total retinal thickness by age. However, in the analysis of separate layers, the IPL, ONL, and PR layers were significantly thinner with age. In the more peripheral inner and outer rings, the overall thickness of the retina and some specific layers (GCL, IPL, and ONL) also significantly decreased with age.

**Table 1.** Mean Macular Thickness and Volume by Retinal Layer

	Retinal Layers										
						Inner		Outer			Retinal
		Inner	Outer	Nerve	Ganglion	Plexi-	Inner	Plexi-	Outer	Photo-	Pigment
	Total	Retinal	Retinal	Fiber	Cell	form	Nuclear	form	Nuclear	receptor	Epithe-
	Retina	Layers	Layers	Layer	Layer	Layer	Layer	Layer	Layer	Layer	lium
Macular thickness (µm)											
Central Circle	277 (34)	188 (26)	86 (14)	13 (3)	16 (8)	21 (4)	23 (7)	28 (6)	89 (13)	68 (5)	18 (14)
Inner Ring											
Nasal	333 (23)	252 (20)	80 (5)	23 (4)	46 (7)	39 (4)	40 (4)	35 (7)	70 (12)	65 (3)	15 (4)
Temporal	318 (21)	237 (19)	80 (8)	19 (2)	42 (8)	38 (4)	37 (4)	31 (4)	72 (9)	65 (3)	15 (7)
Superior	329 (22)	248 (19)	79 (4)	27 (5)	47 (6)	37 (4)	39 (4)	35 (8)	65 (12)	64 (2)	15 (4)
Inferior	325 (21)	246 (20)	79 (7)	26 (4)	46 (8)	37 (4)	39 (4)	35 (8)	65 (12)	64 (2)	15 (6)
Average	326 (20)	246 (19)	80 (5)	24 (3)	45 (7)	38 (4)	39 (3)	34 (5)	68 (9)	64 (2)	15 (5)
Outer Ring											
Nasal	299 (20)	221 (19)	77 (3)	53 (10)	32 (4)	25 (3)	31 (3)	28 (3)	53 (9)	64 (2)	13 (2)
Temporal	270 (15)	192 (15)	77 (3)	22 (3)	30 (8)	28 (3)	30 (3)	27 (2)	55 (7)	64 (2)	13 (2)
Superior	283 (16)	204 (15)	78 (3)	41 (8)	29 (4)	24 (3)	30 (4)	26 (3)	56 (7)	65 (2)	14 (3)
Inferior	274 (17)	197 (16)	76 (3)	41 (9)	28 (4)	24 (3)	30 (3)	26 (3)	49 (6)	63 (2)	13 (1)
Average	282 (15)	204 (15)	77 (3)	39 (6)	30 (4)	25 (3)	30 (2)	27 (2)	53 (6)	64 (2)	13 (2)
Macular volume (mm^3^)											
ETDRS grid	8.2 (0.5)	6.0 (0.4)	2.2 (0.1)	1.0 (0.2)	0.9 (0.1)	0.8 (0.1)	0.9 (0.1)	0.8 (0.1)	1.6 (0.2)	1.8 (0.1)	0.4 (0.1)

Numbers are presented as mean (SD).

**Table 2.** Mean Macular Thickness and Volume by Age Group

	Age Groups, Tertile (Years)			
	Tertile 1 69–78	Tertile 2 78–83	Tertile 3 83–101	*P* Value
Total retina, number of eyes	330	321	255	
Macular thickness (µm)				
** **Central circle	278 (33)	277 (28)	277 (42)	0.89
** **Inner ring	330 (20)	326 (18)	323 (23)	0.001
** **Outer ring	284 (16)	280 (15)	280 (15)	0.004
Macular volume (mm^3^)				
** **ETDRS grid	8.3 (0.5)	8.2 (0.4)	8.2 (0.5)	0.002
Inner retinal layers, number of eyes	312	307	236	
Macular thickness (µm)				
** **Central circle	190 (28)	188 (23)	187 (26)	0.25
** **Inner ring	249 (19)	245 (17)	241 (19)	<0.0001
** **Outer ring	206 (15)	203 (14)	201 (14)	0.001
Macular volume (mm^3^)				
** **ETDRS grid	6.1 (0.4)	6.0 (0.4)	5.9 (0.4)	<0.0001
Outer Retinal Layers, number of eyes	330	321	255	
Macular thickness (µm)				
** **Central circle	86 (11)	86 (12)	86 (20)	0.40
** **Inner ring	80 (5)	79 (3)	80 (7)	0.98
** **Outer ring	77 (3)	77 (2)	78 (3)	0.10
Macular volume (mm^3^)				
** **ETDRS grid	2.2 (0.1)	2.2 (0.1)	2.2 (0.1)	0.57
Nerve fiber layer, number of eyes	319	309	241	
Macular thickness (µm)				
** **Central circle	13 (3)	13 (3)	13 (2)	0.32
** **Inner ring	23 (3)	24 (3)	24 (3)	0.21
** **Outer ring	39 (6)	39 (6)	39 (7)	0.83
Macular volume (mm^3^)				
** **ETDRS grid	1.0 (0.1)	1.0 (0.2)	1.0 (0.2)	0.93
Ganglion cell layer, number of eyes	317	309	240	
Macular thickness (µm)				
** **Central circle	17 (13)	16 (4)	16 (4)	0.08
** **Inner ring	46 (7)	45 (6)	44 (7)	<0.0001
** **Outer ring	30 (4)	30 (4)	29 (4)	<0.0001
Macular volume (mm^3^)				
** **ETDRS grid	0.9 (0.1)	0.9 (0.1)	0.9 (0.1)	<0.0001
Inner plexiform layer, number of eyes	307	308	230	
Macular thickness (µm)				
** **Central circle	22 (4)	21 (4)	21 (4)	0.04
** **Inner ring	38 (4)	37 (4)	37 (4)	<0.0001
** **Outer ring	26 (2)	25 (3)	25 (2)	0.006
Macular volume (mm^3^)				
** **ETDRS grid	0.80 (0.07)	0.78 (0.07)	0.78 (0.07)	0.0004
Inner nuclear layer, number of eyes	307	308	230	
Macular thickness (µm)				
** **Central circle	22 (7)	23 (6)	24 (7)	0.10
** **Inner ring	39 (4)	39 (3)	39 (3)	0.03
** **Outer ring	30 (3)	30 (2)	30 (2)	0.13
Macular volume (mm^3^)				
** **ETDRS grid	0.9 (0.1)	0.9 (0.1)	0.9 (0.1)	0.08
Outer plexiform layer, number of eyes	311	311	234	
Macular thickness (µm)				
** **Central circle	28 (6)	28 (7)	28 (6)	0.61
** **Inner ring	34 (4)	34 (5)	34 (4)	0.89
** **Outer ring	27 (2)	27 (2)	27 (2)	0.16
Macular volume (mm^3^)				
** **ETDRS grid	0.8 (0.1)	0.8 (0.1)	0.8 (0.1)	0.27
Outer nuclear layer, number of eyes	311	311	234	
Macular thickness (µm)				
** **Central circle	90 (12)	90 (12)	87 (15)	0.01
** **Inner ring	69 (8)	68 (9)	65 (10)	0.0001
** **Outer ring	54 (6)	53 (7)	52 (6)	0.003
Macular volume (mm^3^)				
** **ETDRS grid	2 (0.2)	2 (0.2)	2 (0.2)	0.001
Photoreceptor layer, number of eyes	330	321	255	
Macular thickness (µm)				
** **Central circle	69 (5)	68 (5)	68 (4)	0.004
** **Inner ring	64 (2)	64 (2)	64 (2)	0.65
** **Outer ring	64 (2)	64 (2)	64 (2)	0.34
Macular volume (mm^3^)				
** **ETDRS grid	1.8 (0.1)	1.8 (0.1)	1.8 (0.1)	0.83
Retinal pigment epithelium, number of eyes	330	321	255	
Macular thickness (µm)				
** **Central circle	17 (10)	18 (11)	18 (20)	0.59
** **Inner ring	15 (5)	15 (3)	15 (6)	0.77
** **Outer ring	13 (2)	13 (1)	13 (2)	0.08
Macular volume (mm^3^)				
** **ETDRS grid	0.4 (0.1)	0.4 (0.04)	0.4 (0.1)	0.25

Numbers are presented as mean (SD).

### Macular Thickness by AMD Presence and Severity

The thickness of the total retina was similar in eyes with and without AMD in the central circle, as well as inner and outer rings (Fig. 2B and Table 3). Eyes with AMD were further classified into early AMD (n = 205), intermediate AMD (n = 185), and late AMD (n = 23). Of the 23 eyes with late AMD, five (22%) had geographic atrophy (both central and noncentral) and 18 (78%) had neovascular AMD. The thickness of the total retina did not vary by AMD severity in the central circle, as well as inner and outer rings (*P* = 0.35, *P* = 0.26, and *P* = 0.51). The thickness of the inner retinal layers was also similar across AMD outcomes in the central circle and outer ring (*P* = 0.11 and *P* = 0.25) but decreased significantly with increasing AMD severity in the inner ring (*P* = 0.003). The thickness of the outer retinal layers decreased significantly with increasing AMD severity in the central circle, as well as inner and outer ring (*P* = 0.009, *P* = 0.002, and *P* = 0.009). In the analysis of the individual retinal layers, the thickness of the RPE increased with AMD severity within the central circle, as well as inner and outer rings (*P* = 0.001, *P* = 0.0005, and *P* = 0.002). These results were likely due to 78% of late AMD eyes being neovascular compared to 22% having geographic atrophy. The PR layer in the central circle, but not the inner and outer rings, was thinner in eyes with AMD compared to those without AMD (*P* = 0.0008). The thickness of the GCL decreased by AMD severity in the inner ring (*P* = 0.05).

**Table 3.** Mean Macular Thickness and Volume by AMD Outcome, Age Adjusted

	AMD				
	No AMD	With AMD			*P* Value
Total retina, number of eyes	486	413			
Macular thickness (µm)					
** **Central circle	277 (2)	278 (2)			0.60
** **Inner ring	327 (1)	326 (1)			0.34
** **Outer ring	282 (1)	281 (1)			0.35
Macular volume (mm^3^)					0.27
** **ETDRS grid	8.2 (0.02)	8.2 (0.02)			
	AMD				
	No AMD	Early AMD	Intermediate AMD	Late AMD	*P* Value

Total retina, number of eyes	486	205	185	23	
Macular thickness (µm)					
** **Central circle	277 (2)	276 (2)	279 (3)	285 (17)	0.35
** **Inner ring	327 (1)	326 (1)	327 (1)	317 (6)	0.26
** **Outer ring	282 (1)	281 (1)	282 (1)	278 (2)	0.51
Macular volume (mm^3^)					
** **ETDRS grid	8.2 (0.02)	8.2 (0.03)	8.3 (0.03)	8.1 (0.08)	0.41
Inner retinal layers, number of eyes	457	194	177	21	
Macular thickness (µm)					
** **Central circle	189.1 (1.3)	189.1 (1.6)	189.0 (1.8)	165.0 (8.1)	0.11
** **Inner ring	246.5 (0.9)	245.8 (1.1)	245.5 (1.1)	223.4 (4.6)	0.003
** **Outer ring	203.7 (0.7)	203.3 (0.9)	203.8 (0.8)	197.1 (2.3)	0.25
Macular volume (mm^3^)					
** **ETDRS grid	6.02 (0.02)	5.99 (0.03)	6.01 (0.02)	5.71 (0.08)	0.05
Outer retinal layers, number of eyes	486	205	185	23	
Macular thickness (µm)					
** **Central circle	84.9 (0.4)	84.1 (0.4)	87.2 (0.9)	119.7 (16.0)	0.009
** **Inner ring	79.0 (0.2)	78.7 (0.2)	80.2 (0.3)	93.1 (5.7)	0.002
** **Outer ring	77.2 (0.1)	77.1 (0.1)	77.6 (0.2)	79.6 (1.2)	0.009
Macular volume (mm^3^)					
** **ETDRS grid	2.20 (0.004)	2.19 (0.005)	2.22 (0.006)	2.37 (0.071)	0.002
Nerve fiber layer, number of eyes	467	198	175	23	
Macular thickness (µm)					
** **Central circle	13.2 (0.2)	13.2 (0.2)	13.1 (0.2)	13.8 (0.7)	0.81
** **Inner ring	23.8 (0.2)	23.7 (0.2)	23.2 (0.2)	23.7 (0.6)	0.07
** **Outer ring	39.2 (0.4)	39.0 (0.4)	38.6 (0.4)	40.7 (1.3)	0.64
Macular volume (mm^3^)					
** **ETDRS grid	0.99 (0.01)	0.98 (0.01)	0.97 (0.01)	1.02 (0.03)	0.42
Ganglion cell layer, number of eyes	466	197	175	22	
Macular thickness (µm)					
** **Central circle	16.5 (0.5)	16.4 (0.4)	16.0 (0.3)	16.7 (1.2)	0.53
** **Inner ring	45.4 (0.3)	45.1 (0.4)	45.0 (0.4)	41.8 (1.4)	0.05
** **Outer ring	29.6 (0.2)	29.7 (0.4)	29.9 (0.2)	28.4 (0.7)	0.88
Macular volume (mm^3^)					
** **ETDRS grid	0.92 (0.01)	0.92 (0.01)	0.93 (0.01)	0.85 (0.03)	0.33
Inner plexiform layer, number of eyes	461	193	175	12	
Macular thickness (µm)					
** **Central circle	21.3 (0.2)	21.4 (0.3)	21.5 (0.3)	21.6 (1.4)	0.39
** **Inner ring	37.6 (0.2)	37.5 (0.2)	37.5 (0.2)	36.4 (0.9)	0.39
** **Outer ring	25.3 (0.1)	25.2 (0.1)	25.3 (0.1)	25.2 (0.4)	0.63
Macular volume (mm^3^)					
** **ETDRS grid	0.79 (0.004)	0.79 (0.004)	0.79 (0.004)	0.78 (0.013)	0.58
Inner nuclear layer, number of eyes	461	193	175	12	
Macular thickness (µm)					
** **Central circle	22.5 (0.3)	23.5 (0.5)	23.4 (0.6)	23.2 (2.3)	0.09
** **Inner ring	38.7 (0.2)	38.7 (0.2)	38.8 (0.3)	37.4 (0.9)	0.91
** **Outer ring	30.1 (0.1)	30.2 (0.1)	30.2 (0.2)	30.4 (0.5)	0.45
Macular volume (mm^3^)					
** **ETDRS grid	0.9 (0.004)	0.9 (0.004)	0.9 (0.005)	0.9 (0.02)	0.54
Outer plexiform layer, number of eyes	466	194	178	14	
Macular thickness (µm)					
** **Central circle	27.9 (0.3)	28.7 (0.5)	28.3 (0.5)	26.3 (1.9)	0.53
** **Inner ring	33.7 (0.2)	33.8 (0.3)	34.1 (0.3)	33.7 (1.0)	0.27
** **Outer ring	26.8 (0.1)	26.8 (0.1)	26.9 (0.1)	27.1 (0.8)	0.52
Macular volume (mm^3^)					
** **ETDRS grid	0.802 (0.003)	0.801 (0.004)	0.807 (0.005)	0.804 (0.020)	0.46
Outer nuclear layer, number of eyes	466	194	178	14	
Macular thickness (µm)					
** **Central circle	89.5 (0.7)	87.8 (1.0)	90.1 (0.9)	73.7 (5.6)	0.17
** **Inner ring	67.9 (0.5)	67.8 (0.6)	68.2 (0.6)	53.3 (3.6)	0.08
** **Outer ring	53.1 (0.3)	53.5 (0.4)	53.8 (0.4)	49.7 (1.2)	0.45
Macular volume (mm^3^)					
** **ETDRS grid	1.6 (0.01)	1.6 (0.01)	1.6 (0.01)	1.5 (0.05)	0.65
Photoreceptor layer, number of eyes	486	205	185	23	
Macular thickness (µm)					
** **Central circle	68.6 (0.2)	67.8 (0.3)	67.5 (0.3)	66.8 (1.6)	0.0008
** **Inner ring	64.4 (0.1)	64.1 (0.1)	64.2 (0.1)	65.0 (1.0)	0.52
** **Outer ring	64.0 (0.01)	63.9 (0.1)	64.1 (0.1)	63.8 (0.4)	0.75
Macular volume (mm^3^)					
** **ETDRS grid	1.82 (0.003)	1.81 (0.004)	1.81 (0.004)	1.82 (0.014)	0.47
Retinal pigment epithelium, number of eyes	486	205	185	23	
Macular thickness (µm)					
** **Central circle	16.1 (0.2)	16.3 (0.2)	19.9 (0.9)	53.1 (16.0)	0.001
** **Inner ring	14.5 (0.1)	14.7 (0.1)	16.0 (0.3)	28.5 (5.6)	0.0005
** **Outer ring	13.2 (0.1)	13.2 (0.1)	13.5 (0.1)	16.0 (1.3)	0.002
Macular volume (mm^3^)					
** **ETDRS grid	0.38 (0.002)	0.385 (0.002)	0.403 (0.004)	0.564 (0.070)	0.0003

Numbers are presented as least squared mean (SE).

### Macular Thickness and Visual Acuity

Mean ± SD BCVA with each age tertile was 82.5 ± 7.9, 80.1 ± 11.9, and 76.5 ± 15.4; BCVA significantly declined with age (*P* < 0.0001), and the presence of late AMD (*P* < 0.0001) when adjusted for age. The association of RPE thickness to BCVA was no longer significant (*P* = 0.61), after excluding women with late AMD. In the age-adjusted model, increased thickness of the ONL was associated with better visual acuity whereas increased thickness of the RPE was associated with reduced BCVA (Table 4). The association of ONL thickness to BCVA weakened but remained statistically significant after excluding patients with cataract. Further excluding participants with late AMD attenuated the association further. The beta-coefficients (*P* values) for the association of ONL thickness to BCVA were 0.096 (0.001) with age-adjustment, 0.070 (0.01) after excluding women with cataract, and 0.047 (0.08) after further excluding women with late AMD.

**Table 4.** Association Between Retinal Layer Thickness and Best Corrected Visual Acuity

			Eye Without		Eyes Without Cataract	
	All Eyes		Cataract		and Late AMD	
Retinal Layers, (µm)	N	Beta Estimate	N	Beta Estimate	N	Beta Estimate
Nerve fiber layer	865	−0.128	730	−0.061	710	−0.053
Ganglion cell layer	863	0.008	730	0.010	710	0.004
Inner plexiform layer	818	0.108	691	0.125	680	0.112
Inner nuclear layer	818	−0.035	691	−0.027	680	−0.030
Outer plexiform layer	824	0.040	697	0.041	684	0.034
Outer nuclear layer	824	0.096^a^	697	0.079^a^	684	0.047
Photoreceptor layer	865	−0.023	730	−0.067	710	−0.040
Retinal pigment epithelium	865	−0.062	730	−0.061	710	−0.024

a *P* < 0.05.

## Discussion

The histologic correlates of OCT images have been studied extensively,^17^^–^^19^ and results of histological^20^^–^^22^ and OCT studies^23^^–^^25^ indicate that many regions of the macula thin with age and neurodegenerative diseases. In the current analysis, we provide normative data on the macular thickness of women age 69 to 101 years and describe the variations in macular thickness across age and AMD outcomes. Results of this report corroborate other population-based studies demonstrating reduced retinal thickness with age, particularly outside the central circle.^5^^–^^8^^,^^26^

The difference in outer retinal thickness between AMD and non-AMD eyes was expected considering AMD is a disease primarily affecting the RPE. However, the difference in thickness between no AMD and early AMD was not discernible, which makes this measurement inadequate for identifying the transition. Outer retinal quantification in advanced AMD is an important biomarker for disease monitoring.^27^^,^^28^ Our data showed increasing outer retinal thickness in all three circles from early to intermediate AMD indicating the role of quantitative monitoring of disease severity.

The positive association between the thickness of the ONL and BCVA, adjusted for age, is noteworthy. Previously, individual retinal layers and impact on visual acuity has mostly been evaluation in relation to epiretinal membrane surgery or other retinal diseases such diabetes and glaucoma.^29^^–^^31^ Histopathologic studies have shown degeneration of ONL with loss of nuclei due to aging.^32^ The current study shows a thinning of ONL with age and that the age-adjusted association between ONL and BCVA persists after excluding participants with cataract, indicating that vision is preserved in those with delayed aging effects on retinal layers. This is explained, only in part, by the presence late AMD in some women.

Vital considerations when evaluating retinal thickness include the accuracy of the SD-OCT segmentation algorithms applied and the boundaries of the individual retinal layers used.^33^ Our study used the Heidelberg Spectralis SD-OCT device and its proprietary segmentation software. We noted errors in the automated segmentation that required manual correction throughout most scans (>80%). Errors in boundary lines, particularly ILM can occur because of commonly seen age-related pathologies, such as epiretinal membranes and thickened vitreous. The graders ensured that the line followed the ILM and made corrections as needed. The boundaries for Heidelberg's segmentation software differ from other devices and algorithms, such as those performed by Cirrus (Carl Zeiss Meditex, Dublin, CA, USA).^34^ These discrepancies likely contribute to the variation in measurements across population-based studies, even among similar population subgroups. For example, despite a similar cohort of elderly Caucasian individuals, our results differed slightly from those obtained by the Beaver Dam Eye Study, which used Topcon 3D-OCT software.^7^

Other factors that likely contribute to differences in retinal thickness measurements across population-based studies include the differences in thickness by gender^5^^–^^7^^,^^35^ and race.^36^^,^^37^ For example, the Beaver Dam Eye Study population (also predominantly Caucasian) was not gender specific,^7^ whereas the CAREDS2 population consisted of only women. We observed thinning of the total retina with increasing age, mainly because of reduced thickness of the inner retinal layers, particularly the GCL, IPL, and ONL. Thinning of these layers was only observed in the inner (3 mm diameter) and outer (6 mm diameter) rings and not in the central circle (1 mm diameter). Our results corroborate other population-based studies demonstrating reduced retinal thickness with age, particularly outside the central circle.^5^^–^^7^^,^^35^

Our study has some limitations. The CAREDS cohort includes only women, of primarily (97%) white race, and higher levels of socioeconomic status than similarly aged women in the American population. For this reason the results may lack generalizability to men, and to women of other races and socioeconomic status (Mares et al., unpublished data). Another limitation is the cross-sectional study design, which limits inferences about causal relationships between the observed associations. In addition, we did not control for axial length and refractive error, whereas previous studies reported that both axial length and refractive errors can influence the macular thickness measurements, particularly in eyes with high axial lengths.^5^^–^^7^^,^^35^^,^^38^^,^^39^ The mean axial length in the population was 23.6 (SD 1.1) and percentage of women with axial length >26 mm was small at 3% and not expected to affect study data. In addition, unlike other SD-OCT devices, the Heidelberg software applies model eye parameters to correct for ocular magnification.^40^ Last, we did not control for other diseases that may affect thickness measurements, such as neurodegenerative diseases.^4^

Despite these limitations, our study has several strengths. CAREDS2 involved a unique and well-characterized cohort of older women, for the study of age-related changes in the eye. An important strength is the age range of CAREDS2 study participants, with few studies containing participants with a mean age of 83 years and age range up to 101 years. We did manual editing of all segmented layers throughout the entire ETDRS grid, which provides the most accurate retinal thickness measurements. In addition, expert graders evaluated each individual retinal layer for confounding pathology that may alter the thickness measurements.

## Conclusions

We provide normative macular thickness data for a unique and well-characterized cohort of women, mostly white, aged 69 to 101 years in the CAREDS2 study, an ancillary study of the WHI-OS. The peripheral macula thinned with increasing age, particularly in the GCL, IPL, and ONL of the inner retina. Retinal thickness did not differ between eyes with AMD compared to without AMD in the neural retina (retina without the RPE layer). Greater thickness in the ONL may confer advantages for visual acuity, independent of age and AMD. Additional studies of retinal thickness in relation to vision function could enhance our understanding of the value in retinal thickness measurements in clinical practice.

With the population aging in the United States and a likely increase in the prevalence of age-related diseases, including AMD, our study will help to better understand AMD among older women. In addition, the results of the current analysis can be used in future epidemiological and clinical studies evaluating the associations between the macular thickness and age-related diseases.
